# How Presenteeism Shaped Teacher Burnout in Cyberbullying Among Students During the COVID-19 Pandemic

**DOI:** 10.3389/fpsyg.2021.745252

**Published:** 2021-10-21

**Authors:** Paula da Costa Ferreira, Alexandra Barros, Nádia Pereira, Alexandra Marques Pinto, Ana Margarida Veiga Simão

**Affiliations:** Research Center for Psychological Science, Faculty of Psychology, University of Lisbon, Lisbon, Portugal

**Keywords:** presenteeism, cyberbullying, burnout, teacher bystanders, COVID-19 pandemic

## Abstract

The pandemic caused by SARS-CoV2 has had an impact on the education sector, and its stakeholders, such as teachers who had to do remote work from their home, despite many constraints. These professionals tried to perform their teaching functions, despite having to deal with adverse situations, such as cyberbullying among their students, as well as their difficulties related to presenteeism and burnout. In this context, this study aimed to understand whether observing cyberbullying among students can be associated with teachers’ productivity loss due to presenteeism and burnout. This study also proposed to examine the role of productivity loss due to presenteeism in the relationship between observing cyberbullying situations among students and teacher burnout. A random sample of 1,044 (*Mage*=51.05, *SD*=7.35; 76.6% female) middle school and high school teachers answered an inventory about their experience working at home during the COVID-19 pandemic, specifically with regards to cyberbullying incidents they observed among their students, their productivity loss due to presenteeism, and their burnout levels. Results from structural equation modeling revealed that observing students engaging in cyberbullying situations was positively associated with productivity loss due to presenteeism and teacher burnout. Also, teacher’s productivity loss due to presenteeism mediated the relationship between observing cyberbullying incidents among their students and their burnout levels. Specifically, the effect of productivity loss due to presenteeism explained the effect of observing cyberbullying incidents on teachers’ burnout levels. These results are innovative and shed light on the importance of teacher wellbeing at their job in the midst of a pandemic, namely, when they observe their students engaging in hostile situations, which may lead them to greater levels of burnout.

## Introduction

The pandemic caused by SARS-CoV2 forced most sectors to go through a process of adaptation to emerging situations in a context that generated uncertainty (Flores et al., 2021). The emergency situation inevitably affected the education sector and those involved in the learning/teaching process (Joshi et al., 2020). Teachers performed their functions, despite the difficulties in adapting to and managing the situation. This adaptation process unleashed a series of harmful psychosocial risks which may have impaired the physical and psychological wellbeing of teachers (Prado-Gascó et al., 2020). In the work context, these risks refer to social, organizational, and work management aspects that can cause physical and/or psychological harm to individuals, such as stress, burnout, or depression (European Center for Disease Prevention and Control, 2020), and have an impact on organizations and, consequently, on the economy (Bailey et al., 2015). A recent UNESCO report (Dorcet et al., 2020) has emphasized the need to address teachers’ wellbeing and the disturbances which may affect their work and which have emerged during the pandemic, such as organizational commitment affecting burnout (Sokal et al., 2021) and positive attitudes toward change, perceptions of principal support, teaching efficacy, and attitudes toward technology negatively predicting burnout (Sokal et al., 2020). Accordingly, in the case of teachers, we propose that observing cyberbullying incidents among students can be considered a psychosocial risk that may be associated with these professionals’ overall wellbeing, since it is a social aspect of their work context which teachers may have to manage.

Cyberbullying proliferated during the COVID-19 pandemic, leading to unhealthy behavior and carrying grave consequences for those involved (Barlett et al., 2021). Considering the challenges presented by the pandemic, we aim to understand whether experiencing cyberbullying as a bystander can be associated with teachers’ productivity loss due to presenteeism and burnout. That is, whether observing these incidents could be related to how teachers may underperform during work due to physical or psychological complications and burnout. Presenteeism is a problem of workers not working at work due to illness, injury, or other condition (Koopman et al., 2002; Johns, 2010). Presenteeism may be impacted by fatigue, low supervisor support, the lack of performance recognition, and inappropriate administration efforts (Dudenhöffer et al., 2017), situations which emerged during the pandemic (Joshi et al., 2020). Even though they are at work, they may not be able to fully perform their duties and are more likely to make mistakes at work and reveal a loss of productivity. Lastly, productivity loss due to presenteeism has been known to be correlated with teachers’ levels of burnout (Ferreira and Martinez, 2012). Thus, we intend to investigate the role of this variable in the relationship between observing cyberbullying among students and teachers’ burnout. By reaching these objectives, this study provides a contribution to the field of presenteeism (Koopman et al., 2002) and to the Job Demands-Resources theory (Bakker and Demerouti, 2007), by introducing a variable from experimental social psychology, such as being a bystander of cyberbullying (Latané and Darley, 1970). In fact, an integrative approach of possible causes and consequences of presenteeism is crucial to understand it (Johns, 2010). Thus, we propose that being a bystander of cyberbullying may be a predecessor of presenteeism as a job demand, since job demands may elicit presenteeism (Miraglia and Johns, 2016), whereas burnout may be a consequent also due to do greater job demands within the context of confinements due to SARS-CoV2.

It is essential to investigate the psychosocial risks emerging for teachers in the context of distance learning forced by confinement because an effective prevention of these types of risks can promote wellbeing at work (Hammer et al., 2019) and increase productivity (Bakker and Wang, 2019). In emergency situations, such as confinements due to SARS-CoV2, there can be an increase in psychosocial risks, such as interpersonal conflicts between teachers, school, students, and even family members (Kubik et al., 2018). Accordingly, these conflicts have been associated with psychological health problems, such as depression, so it is imperative to understand whether, in the digital sphere, this type of phenomenon occurred in this situation. Since teachers during the confinement were at home working, we believe that these psychosocial risks became part of their job demands (Bakker and Demerouti, 2007), since they involve extra effort and skills to perform their job accordingly.

Teachers play a key role in solving peer aggression because they can witness the development of many interpersonal relationships among adolescents in the classroom (DeSmet et al., 2015) and even mobilize efforts to prevent this phenomenon (Eden et al., 2013). However, evidence has shown that in face-to-face settings, teachers tend to notice bullying situations more than cyberbullying, interpret the first more as an emergency, take responsibility for intervening, know the appropriate form of action, and provide assistance (Eldridge and Jenkins, 2020). From this evidence, teachers’ capacity to affectively empathize with cyberbullying victims was positively associated with taking responsibility for intervening in cyberbullying situations. Thus, it is crucial to investigate the role of teachers as bystanders of aggression among peers, such as cyberbullying. Bystanders experience several cognitive and behavioral processes when facing critical situations, such as noticing something is wrong, interpreting the severity of the situation, assuming responsibility for intervening (or not), deciding on the appropriate form of assistance, and intervening (or not; Latané and Darley, 1970). In this study, we focus specifically on bystanders of cyberbullying due to the fact that it is often more difficult for teachers to identify this phenomenon (Eldridge and Jenkins, 2020), and also, because it has proliferated with the succession of lockdowns due to SARS-CoV2 (Barlett et al., 2021). Specifically, cyberbullying is the act of deliberately and repeatedly posting or sending harmful messages or engaging in other forms of social aggression among peers while using digital technologies with the aim of hurting someone (Belsey, 2005; Hinduja and Patching, 2009).

Some evidence has shown that female teachers tend to demonstrate greater concern about cyberbullying, have more information on the subject, and believe more in the school’s commitment to deal with the problem than male teachers (DeSmet et al., 2015). Nonetheless and in general, teachers have reported that they lack training, skills, and confidence to deal with the problem (Li, 2009). In fact, we consider that this lack of training, perceived skills, and confidence to deal with cyberbullying situations falls within the lack of resources proposed by Bakker and Demerouti (2007; as conceptualized by the Job Demands-Resources theory), since these variables are important aspects that may impede teachers’ regular functioning at their job. According to the Job Demands-Resources theory, job demands (e.g., such as observing cyberbullying) may lead to less engagement and more burnout, whereas resources (e.g., training in how to deal with cyberbullying) can lead to less burnout and more engagement at work, which in turn, affect job performance (Bakker and Demerouti, 2007) and a decreased productivity loss due to presenteeism (Ferreira et al., 2019). The strain that is associated with excessive job demands and reduced work engagement may give rise to presenteeism, especially when there are little work resources (McGregor et al., 2016). Moreover, although adolescents may not consider teachers as part of the solution to preventing cyberbullying (Mishna et al., 2014), it appears that when they report incidents to their teachers, their perceptions of the school climate improve significantly (Veiga Simão et al., 2017).

Cyberbullying can have harmful consequences for the mental health of individuals, as it can negatively influence their relationships and social reputation, which, in turn, contributes to a decrease in their wellbeing (Anderson and Sturm, 2007). Accordingly, cyberbullying can also be considered a public health problem and should be the responsibility of public health systems and services. Research has indicated that victims of cyberbullying tend to be at greater risk of developing aggressive, depressive, and somatic symptoms (Gradinger et al., 2009). However, recent evidence has shown that cyberbullying bystanders can also reveal greater levels of depression, anxiety, and somatic symptoms and those who have not been exposed to the phenomenon (Doumas and Midgett, 2020). Although these studies have used samples with children and adolescents, some of the literature suggests that teachers themselves can also get involved in online aggression situations. Recent studies point to the cybervictimization of teachers by guardians (Küçüksüleymanoğlu, 2019) and even by students (Kyriacou and Zuin, 2015), despite the fact that the phenomenon of cyberbullying is characterized by being among peers. Male teachers have also shown greater involvement as cyberaggressors than female teachers (Tosun, 2016), although they do not report its occurrence on a large scale, as this type of behavior is not considered adjusted or accepted according to social norms. Following these results, other studies have highlighted the negative effects that cyberbullying can have on teachers at an emotional, physiological, and behavioral level (Kopecký and René, 2016). In line with the evidence presented on cyberbullying as a potential psychosocial risk and consequent job demand, as well as on the impact the phenomenon may have on children and adolescent bystanders and teachers, we question as:

Can observing cyberbullying among students be associated with teachers’ productivity loss due to presenteeism and burnout?

Psychosocial risks have been associated with physical and mental health problems, such as lack of motivation and reduced effectiveness at work, which in the area of teaching can have an impact on the quality of teaching (Bergh et al., 2018). Psychosocial risks have been associated with physical and psychological health problems (Bergh et al., 2018), namely, work-related stress and a reduction in social interaction (Junne et al., 2018), burnout (Maslach et al., 2001), depression and anxiety, lack of concentration (Nielsen et al., 2020), and low job satisfaction (Guadix et al., 2015).

In this study, we focus on the specific context of cyberbullying, a psychosocial risk, and consequent job demand teachers may face when working with students online, as was the case with the lockdowns due to SARS-CoV2. Moreover, considering that previous studies have shown that psychological conditions, such as high levels of stress and lack of emotional fulfillment, can impact the existence of presenteeism (Boles et al., 2004; Pelletier et al., 2004), as is the case with cyberbullying situations, we proposed to consider teachers’ productivity loss due to presenteeism.

Although there are several definitions of presenteeism in the literature (Johns, 2010), all recent perspectives agree that it essentially consists of being present at work, despite feeling unhealthy. Symptoms of presenteeism include various types of medical conditions, such as migraines and other types of episodic or chronic pain, allergies, asthma, dermatitis, anxiety, and depression (Koopman et al., 2002), or even other types of distracting events (Hummer et al., 2002). Presenteeism is often associated with significant losses in productivity, as it hinders the quality of professional life and increases the perception of ineffectiveness at work (Lofland et al., 2004). It appears in most professions but occupies a particularly high place among the education and health sectors (Aronsson et al., 2000; Ferreira and Martinez, 2012); therefore, we opted to examine this variable in our study.

Professions with relational contact with others also tend to increase levels of burnout, as is the case with teaching. In fact, there is scientific evidence indicating that teachers have more burnout than other professionals, such as mental health professionals, domestic, and personal care professionals (Shirom and Ezrachi, 2003). Burnout has been defined by some of the literature as a prolonged response to stressful, emotional, and interpersonal situations at work (Maslach et al., 2001), and thus, representing a lack of energy. It can include three distinct dimensions or phases (Maslach et al., 1986), namely (1) emotional exhaustion (2) professional effectiveness, and (3) depersonalization. Accordingly, emotional exhaustion can refer to a feeling of emotional and physical exhaustion. Teachers can feel exhausted due to work overload. Professional effectiveness can refer to feelings of (failure) and (lack of) competence. Depersonalization may be associated with the lack of personal responses and the absence of feelings toward others. On the other hand, burnout can be considered as the degree of physical and psychological fatigue and exhaustion that is perceived by the self as related to their work (Kristensen et al., 2005). Furthermore, it has also been described as emotional exhaustion, physical fatigue, and cognitive weariness (Melamed et al., 1992; Shirom and Melamed, 2006; Gerber et al., 2018).

High demands and lack of resources can lead to a series of negative consequences for workers (Karasek, 1979), namely, psychosomatic health problems and burnout. Previous studies have shown that primary school teachers tend to be less emotionally exhausted and depersonalized, and more professionally effective, than secondary school teachers and that older teachers have higher levels of emotional exhaustion (Russell et al., 1987; Byrne, 1991). Since the sample we worked with in this study presented a mean age of over 50, we opted to focus specifically on the emotional exhaustion dimension of burnout, which is the central feature of the construct, as some of the literature indicates (Kristensen et al., 2005). Accordingly, these teachers’ emotional exhaustion (23.6% of the variance explained) was significantly predicted by presenteeism variables (our mediating construct), unlike professional effectiveness, which was not, and cynicism which only explained 5.6% of the variance (Ferreira and Martinez, 2012). Therefore, from now on, we mention burnout as representative of teachers’ emotional exhaustion, as described in previous studies (Kristensen et al., 2005).

Recent research has shown that 15% of educational professionals are at moderate risk of burnout and that the percentage of employees with burnout syndromes increased from 8 to 15% between 2008 and 2013 (Aumayr-Pintar et al., 2020). Due to the high prevalence of burnout in the education sector in recent years, namely, as a risk for teachers (Yerdelen et al., 2016), the study of burnout in the education sector becomes essential (Schonfeld et al., 2019), as it can have a negative impact on teaching (Travers, 2017) in terms of work motivation (McLean et al., 2019), depression (Martínez-Monteagudo et al., 2019), and interpersonal relationships with students (Travers, 2017).

According to previous studies, working while one is ill may lead to burnout (Demerouti et al., 2009) because the risk of underperforming when individuals feel sick at work (Wright and Cropanzano, 1998) may lead them to use performance protection strategies (Hockey, 1993), such as investing more, in order to work as well as healthy workers, as opposed to staying at home sick to minimize their resource losses (Bakker and Demerouti, 2007). Being sick at work may have psycho-physiological consequences (Kivimäki et al., 2005), whereas staying at home can aid physical and psychological recuperation and recovery, as well as wellbeing (Fritz and Sonnentag, 2005). Therefore, if workers do not take the time to regenerate their psycho-physiological state, but rather, go to work while they are sick, they may accumulate more exhaustion and feel burned out because they have used up their energy trying harder to compensate for their exhaustion (Demerouti et al., 2005) and to avoid the loss of resources (Bakker and Demerouti, 2007).

In times of the COVID-19 pandemic, we believe that the same process may have occurred with teachers during the lockdown while they worked at a distance witnessing disturbing events, such as cyberbullying. In other words, we believe that being a bystander of cyberbullying among their students may have led teachers to underperform as they felt unwell while working remotely from their homes, which in turn, may have directed them to use performance protection strategies to compensate for their exhaustion and therefore, leading them to burnout. Hence, we question as:

Can productivity loss due to presenteeism explain the relationship between observing cyberbullying among students and teachers’ burnout?

## Materials and Methods

### Design

This study presents a cross-sectional design, while exploring the relationship between an independent variable (teachers as bystanders of cyberbullying among students), a dependent variable (teacher burnout), and a mediator variable (teachers’ productivity loss due to presenteeism).

### Participants and Procedures

A total of 1,044 teachers working in Portugal participated in this study (*M_age_*=51.05; *SD*=7.35), 76. 6% of whom were female. In terms of teachers’ daily professional activity, 69.4% mentioned they worked more than 6h a day, whereas 30.6% referred that they worked 6 or less hours per day. As for the grade-levels teachers taught, 54.4% taught 7th, 8th, and 9th grades (third cycle in Portugal), 52.4% taught 10th, 11th, and 12th grades (high school in Portugal), and 27.3% taught 5th and 6th grades (second cycle in Portugal). A total of 34.1% of these teachers had an overlap in the cycles they taught. Moreover, 10.4% had up to 10years of teaching experience, 16.1% had between 11 and 20years, 44.6% mentioned they had between 21 and 30years, and 28.9% between 31 and 45years. As for Internet use, 47.8% considered themselves to be very experienced, 45.6% said they were more or less experienced, and 6.6% had little to no experience.

This study was authorized by the ethics committee of the research team’s institution. All participants voluntarily and anonymously responded to an online inventory individually in the second trimester of, 2020. We used self-report measures since they enabled us to gather information about the subjective experiences of teachers as bystanders of cyberbullying (Graham et al., 2003). The instrument was sent by email and a link for access. Our response rate was 100%, since all 1,044 teachers completed the instrument.

### Instruments

#### Teachers as Bystanders of Cyberbullying

An adaptation (i.e., including translation and changes to items and/or instructions to fit the specific context of teachers’ online teaching during the pandemic) of the questionnaire of the observer of the Cyberbullying Inventory (originally created and validated by Francisco et al., 2015) for University Students was used. Teachers were instructed to think about the months of distance learning due to confinement because of COVID-19 and to answer whether they had observed repeated behavior(s) among students with the intention of hurting someone through various platforms, such as the Zoom, Skype, Email, Chat, Messenger, Facebook, YouTube, Blogs, and WhatsApp. The Teachers as Bystanders of Cyberbullying Questionnaire (TBCQ) contains nine items (*α*=0.82) that ask participants to report how often they observed students engaging in cyberbullying situations (e.g., “I saw someone being threatened.”) on a Likert-type scale from 1 (never) to 5 (several times a day). We performed CFA, which presented good values according to the literature (Hooper et al., 2008). Specifically, *χ*^2^(25)=171.74, *p*<0.00, *χ*^2^/*df*=6.87; CFI=0.95; GFI=0.96; IFI=0.92; AIC=211.74; RMSEA=0.07, LO=0.06, HI=0.08; SRMR=0.03.

#### Productivity Loss Due to Presenteeism

An adaptation (i.e., including translation and changes to items and/or instructions to fit the specific context of teachers’ online teaching during the pandemic) of the Productivity Scale due to Presenteeism (originally created and validated by Koopman et al., 2002) was used. Teachers were requested to describe their experiences working as a teacher during confinement because of COVID-19. They were informed that “health problems” could be physical health or mental health, such as “back pain,” “cardiovascular problems,” “constipation,” “stomach pain,” “depression,” or other similar conditions. Participants answered three items (*α*=0.90) of the Productivity Loss due to Presenteeism Scale (PLPS; e.g., “Health problems inhibited me from taking pleasure in work.”) on a Likert-type scale from 0 (no, I never felt sick) to 5 (yes, more than 10 times). We performed CFA, which presented good values for this sample were good in accordance with the literature (Hooper et al., 2008). Specifically, *χ*^2^(4)=14.98, *p*<0.00, *χ*^2^/*df*=3.74; CFI=0.99; GFI=0.99; IFI=0.99; AIC=48.98; RMSEA=0.05, LO=0.02, HI=0.08; SRMR=0.01.

#### Teacher Burnout

An adaptation (i.e., including translation and changes to items and/or instructions to fit the specific context of teachers’ online teaching during the pandemic) of the Copenhagen Burnout Inventory questionnaire (originally created and validated by Kristensen et al., 2005) was used. Teachers were asked to take into account their current situation of distance learning due to mandatory confinement because of COVID-19. Then, they were instructed to answer all the questions presented considering the academic period in which they had to teach in this context. Participants responded to eight items (*α*=0.91) with the Teacher Burnout Questionnaire (TBQ; e.g., “I feel frustrated with my job.”) on a Likert-type scale from 0 (never) to 4 (always). We performed CFA, which presented good values for this sample according to the literature (Hooper et al., 2008). Specifically, *χ*^2^(12)=34.21, *p*<0.00, *χ*^2^/*df*=2.85; CFI=0.99; GFI=0.99; IFI=0.99; AIC=66.21; RMSEA=0.04, LO=0.02, HI=0.05; SRMR=0.01.

### Common Method Variance

In addition, we computed the Harman’s single-factor test to control the potential common method variance due to the self-reported nature of the instruments. Specifically, there is common method variance if a single-factor is extracted (Podsakoff et al., 2013). Therefore, to compute this test, the TBCQ, PLPS, and the TBQ were loaded into a confirmatory factor analysis. A three-factor model [*χ*^2^(149)=853, *p*<0.00, *χ*^2^/*df*=5.72; CFI=0.93; GFI=0.93; IFI=0.93; AIC=935.57; RMSEA=0.06, LO=0.06, HI=0.07; SRMR=0.04] provided better fit indices than a single-factor model [*χ*^2^(152)=4467.47, *p*<0.00, *χ*^2^/*df*=29.39; CFI=0.55; GFI=0.59; IFI=0.55; AIC=4543.47; RMSEA=0.16, LO=0.16, HI=0.16; SRMR=0.16], hence revealing no common method variance (see Table 1 for factor score weights). This evidence corroborates the three distinct constructs that are being assessed. The composite reliability scores were equal to or higher than 0.80 (Hair et al., 2010) for each of the three dimensions (TBCQ=0.83; PLPS=0.89; and the TBQ=0.91), whereas the Average Variance Extracted (AVE) was close or higher than 0.50 (PLPS=0.74 and the TBQ=0.59), and greater than the variance shared with the remaining constructs, hence supporting convergent validity for PLPS and TB (Henseler et al., 2009). The TBCQ revealed lower levels of AVE (0.36), as it is a very distinct theoretical construct from the other two dimensions. Lastly, our findings confirm the variables’ discriminant validity (TBCQ=0.02; PLPS=0.18; and the TBQ=0.18) with all of the Average Shared Variance (ASV) scores below the AVE value (Hair et al., 2010).

**Table 1:** Factor score weights of the three distinct constructs analyzed in this study.

	Factor 1	Factor 2	Factor 3
*Teachers as bystanders of cyberbullying*			
1. I saw someone being threatened.	0.65		
2. I saw someone being harassed with sexual content.	0.38		
3. I saw rumors being spread about someone.	0.72		
4. I saw someone impersonating someone else.	0.51		
5. I saw someone being made fun of.	0.65		
6. I saw someone being insulted.	0.75		
7. I saw someone show that they had information about someone else’s life that could affect their psychological wellbeing.	0.64		
8. I saw someone’s private life data being released.	0.49		
9. I saw someone’s image being used without permission.	0.52		
*Productivity Loss due to Presenteeism*			
1. Due to my health problems, the difficulties that are normally part of my job were more complicated to manage.		0.86	
2. Health problems inhibited me from taking pleasure in work.		0.88	
3. I felt desperate in carrying out certain work tasks due to my health problems.		0.85	
*Teacher Burnout*			
1. I feel exhausted at the end of the workday.			0.83
2. I feel exhausted in the morning thinking that I will have to work.			0.77
3. I feel tired with every hour I work.			0.80
4. I feel my job is more emotionally draining.			0.75
5. I feel frustrated with my work.			0.63
6. I feel exhausted from my work.			0.89
7. I have enough energy for my family and friends during my rest time.			0.69

### Data Analysis Strategy

Before performing structural equation modeling, we computed Pearson correlations. We examined how the relationship being a bystander of cyberbullying and teacher burnout could be mediated by productivity loss due to presenteeism. We assessed the significance of the regression coefficients with IBM AMOS 26 after estimating the parameters through Maximum Likelihood. We used Maximum Likelihood because not only did we work with a large sample size, which reduces any issues regarding multivariate non-normality (Hair et al., 2010), but also it is considered a robust estimator regarding both normally distributed data, as well as any violations of normality assumptions (Bollen, 1989; Diamantopoulos et al., 2000). In fact, Monte-Carlo experiments have provided evidence that no major differences in results from structural equation modeling analysis using the Maximum Likelihood estimator on studies with different sample sizes with different Kurtosis and Skewness levels (Reinartz et al., 2009). Moreover, Bootstrapping methods are increasingly used to resolve these issues (Preacher and Hayes, 2004), which is what we also present in our results section. Then, we assessed the possible significant effects of the control variables age and sex. We tested the significance of the total, direct, and indirect effects with *χ*^2^ tests (Marôco, 2010). We considered effects *p*<0.05 significant. Lastly, we computed the bootstrapping method (2000 samples, CI 90%) to test for mediation effects (Preacher and Hayes, 2008).

## Results

In this section, we present a descriptive analysis and the Pearson correlations between the variables (see Table 2). Results for the general sample revealed a positive significant correlation between all of the variables, therefore, being a bystander of cyberbullying among students is significantly correlated with teachers’ burnout and their productivity loss due to presenteeism, and teachers’ burnout is also positively associated with their productivity loss due to presenteeism.

**Table 2:** Descriptive statistics and correlations between the variables of this study.

Variables	*M*	*SD*	Correlations	
			1	2
1. Teachers as bystanders of cyberbullying	1.12	(0.28)		
2. Productivity loss due to presenteeism	2.26	(0.54)	0.09^**^	
3. Teacher Burnout	3.08	(0.80)	0.15^**^	0.44^**^

** *p<0.01.*

We questioned whether observing cyberbullying among students could be associated with teachers’ productivity loss due to presenteeism and burnout. The correlations presented indicate that in fact, these variables are associated. Moreover, from the analyses done with structural equation modeling, we tested and verified that the predictor variables were positively associated with the dependent variable. Our adjusted structural equation model [*χ*^2^(166)=879.58, *p*<0.05, *χ*^2^/*df*=5.29, CFI=0.93, GFI=0.92, IFI=0.93, RMSEA=0.06, LO=0.06, HI=0.07, AIC=967.58] presented 35% of the variance relating to teachers’ burnout. The standardized total effect of observing cyberbullying behavior among students on teachers’ burnout was 0.16 [CI90, LO=0.09 HI=0.22] and 0.15 [CI90, LO=0.09 HI=0.20] on productivity loss due to presenteeism. Also, the standardized total effect of productivity loss due to presenteeism on teachers’ burnout was 0.57 [CI90, LO=0.53 HI=0.62]. All of these paths were statistically significant according to the Bootstrap sampling method (*p*<0.01).

**Figure 1:**
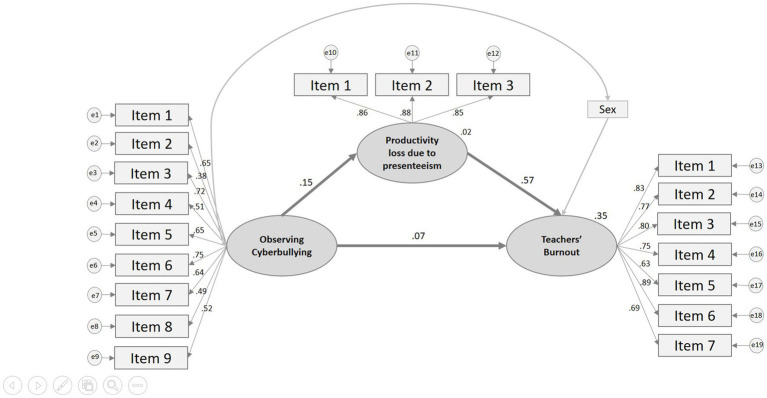
Productivity loss due to presenteeism explaining the relationship between observing cyberbullying incidents and teachers’ burnout. Items for Teachers as bystanders of cyberbullying: 1. I saw someone being threatened; 2. I saw someone being harassed with sexual content; 3. I saw rumors being spread about someone; 4. I saw someone impersonating someone else; 5. I saw someone being made fun of; 6. I saw someone being insulted; 7. I saw someone show that they had information about someone else’s life that could affect their psychological wellbeing; 8. I saw someone’s private life data being released; and 9. I saw someone’s image being used without permission. Items for Productivity Loss due to Presenteeism: 1. Due to my health problems, the difficulties that are normally part of my job were more complicated to manage; 2. Health problems inhibited me from taking pleasure in work; and 3. I felt desperate in carrying out certain work tasks due to my health problems. Items for Teacher Burnout: 1. I feel exhausted at the end of the workday; 2. I feel exhausted in the morning thinking that I will have to work; 3. I feel tired with every hour I work; 4. I feel my job is more emotionally draining; 5. I feel frustrated with my work; 6. I feel exhausted from my work; and 7. I have enough energy for my family and friends during my rest time.

We also questioned whether productivity loss due to presenteeism could explain the relationship between observing cyberbullying among students and teachers’ burnout. Figure 1 shows the conceptual model proposed in this study.

The standardized direct effect of observing cyberbullying behavior among students on teachers’ burnout was 0.07 [CI90, LO=0.01 HI=0.13] and 0.15 [CI90, LO=0.09 HI=0.20] on productivity loss due to presenteeism. Also, the standardized direct effect of productivity loss due to presenteeism on teachers’ burnout was 0.57 [CI90, LO=0.53 HI=0.62]. These trajectories were statistically significant, with the exception of the direct effect of observing cyberbullying incidents on teachers’ burnout according to the Bootstrap sampling method (*p*<0.01). Moreover, the standardized indirect effect of observing cyberbullying behavior among students on teachers’ burnout was 0.08 [CI90, LO=0.06 HI=0.13] and statistically significant according to the Bootstrap sampling method (*p*<0.01). These results reveal how the relationship between observing cyberbullying incidents among students on teachers’ burnout ceases to exist when it is mediated through productivity loss due to presenteeism. Therefore, the mediator variable can explain the relationship between independent and the dependent variable.

We also tested for possible effects of age and sex by controlling these variables because as mentioned in the literature (García-Arroyo et al., 2019), they may affect burnout. Age revealed no significant effect on the dependent or mediator variable. Sex had a significant positive standardized total effect of 0.07 [CI90, LO=0.03 HI=0.12] on burnout only, revealing that female teachers reported more burnout than male teachers, which is consistent with the literature.

## Discussion

During the pandemic caused by SARS-CoV2 teachers performed their work tasks while being exposed to a series of harmful psychosocial risks which may have impaired their physical and psychological wellbeing (Prado-Gascó et al., 2020), such as cyberbullying among their students. This study answered a call by a UNESCO report (Dorcet et al., 2020) for research to focus on the need to address teachers’ wellbeing and the disturbances which may affect their work and which have emerged during the pandemic (Joshi et al., 2020). Hence, we aimed to understand whether observing cyberbullying among students could be associated with teachers’ productivity loss due to presenteeism and burnout. We also aimed to understand whether productivity loss due to presenteeism could explain the relationship between observing cyberbullying among students and teachers’ burnout. We specifically highlighted teachers’ experiences with observed cyberbullying incidents because this phenomenon increased during the COVID-19 pandemic and led to unhealthy behavior and severe consequences for those involved (Barlett et al., 2021).

Our results demonstrated that observing cyberbullying incidents among students was positively related to teachers’ productivity loss due to presenteeism and burnout, which is in line with and adds to previous literature that has provided evidence that cyberbullying can have an impact on teachers at an emotional, physiological, and behavioral level (Kopecký and René, 2016). As bystanders of cyberbullying, teachers may have potentially experienced depression, anxiety, or somatic symptoms and those who have not been exposed to the phenomenon, which corroborates previous studies examining different samples with diverse characteristics (Doumas and Midgett, 2020). Moreover, the positive and significant relationship between observing cyberbullying incidents among students was positively related to teachers’ productivity loss due to presenteeism and burnout may also be interpreted as a function of these professionals’ beliefs that they lack training, skills, and confidence to deal with the phenomenon and those involved (Li, 2009). This lack of training, perceived skills, and confidence to deal with cyberbullying may constitute an important lack of resources (Bakker and Demerouti, 2007), since they may hinder teachers’ regular functioning at their job.

The findings reported in this study also revealed that productivity loss due to presenteeism explained the relationship between observing cyberbullying among students and teachers’ burnout. Specifically, results showed that observing cyberbullying among students could be positively associated with higher levels of teachers’ burnout, but being at work while not feeling well (i.e., physically or psychologically), fully mediated that association, thus reducing the relationship between the independent and dependent variables. This could suggest that how cyberbullying among students is interpreted to the point that it creates burnout in teachers may be dependent on teachers’ perceived inefficiency at work due to their physical and psychological state. Since presenteeism has been known to affect professionals from the education sector severely (Ferreira and Martinez, 2012), it would be no surprise that it could determine the relationship between observing continuous online harassment among their students (i.e., cyberbullying), which could constitute a high job demand with little intervention resources for teachers (Karasek, 1979) and their burnout levels. Moreover, in the particular context of the lockdown due to the COVID-19 pandemic and the student difficulties that may have arisen, teachers worked from home; therefore, the line between what separates absenteeism (i.e., staying home while one is sick) from presenteeism could have been tougher to draw and hence, these professionals may have felt compelled to use performance protection strategies (Hockey, 1993), such as investing more, and meet extraordinary needs which could have triggered a loss of resource (Bakker and Demerouti, 2007). This conjecture may have hindered teachers from physical and psychological recuperation and recovery (Fritz and Sonnentag, 2005). Therefore, being a bystander of cyberbullying among students may have led teachers to underperform as they felt unwell while working remotely from their homes, which in turn, may have directed them to use performance protection strategies to compensate for their exhaustion and thus, lead them to burnout. Ultimately, if teachers observed cyberbullying, then, they were likely to report more burnout. This relationship could be explained by detailing the involvement of productivity loss due to presenteeism. Thus, teachers who reported that they observed cyberbullying, reported feeling burnout, and in turn, those with productivity loss due to presenteeism reported higher levels of burnout.

### Theoretical Contribution

This study provides a theoretical contribution to the literature on productivity loss due to presenteeism (Koopman et al., 2002) and to the Job Demands-Resources theory (Bakker and Demerouti, 2007), by introducing a variable from experimental social psychology, such as being a bystander of cyberbullying (Latané and Darley, 1970). Being a bystander of a harmful phenomenon, such as cyberbullying (Belsey, 2005; Hinduja and Patching, 2009), implies noticing there is an emergency, interpreting the event as such, taking responsibility for intervening (Latané and Darley, 1970), understanding one’s own emotional reactions (Eldridge and Jenkins, 2020), self-efficacy beliefs (Ferreira et al., 2020), and considering the rewards and cost consequences of intervening in specific contexts with others observing (Batson, 1994). Thus, the role of the bystander of cyberbullying carries a heavy load which could add to teachers’ already overloaded job demands (Bakker and Demerouti, 2007), culminating in a psychosocial risk of interpersonal conflict during confinement due to SARS-CoV2 for these professionals (Kubik et al., 2018), even though they are key elements in resolving peer aggression situations (DeSmet et al., 2015; Veiga Simão et al., 2017). Moreover, considering an integrative approach of possible causes and consequences of presenteeism (Johns, 1,010), we found that being a bystander of cyberbullying may be a predecessor of presenteeism as a job demand, because job demands may elicit presenteeism (Miraglia and Johns, 2016), whereas burnout may be a consequent within the context of confinements due to SARS-CoV2.

Moreover, teachers’ lack training, skills, and confidence to deal with cyberbullying (Li, 2009) can be translated as a lack of resources (Bakker and Demerouti, 2007), since these resources are essential to aid teachers’ regular functioning at their job and impede any possible disengagement from it (Demerouti et al., 2001). In fact, as teachers gain awareness of this lack of resources to deal with cyberbullying, and as they may also be impacted negatively by the phenomenon (Doumas and Midgett, 2020), they could be at risk of underperforming (Wright and Cropanzano, 1998), leading them to invest in protection strategies (Hockey, 1993), to minimize their resource losses (Bakker and Demerouti, 2007). Having to work at home due to the pandemic could have hindered teachers from recuperation, recovery, and wellbeing (Fritz and Sonnentag, 2005), and hence, they may have experienced productivity loss due to presenteeism, which led them to more burnout as they tried to compensate for their exhaustion (Demerouti et al., 2005) and avoid the loss of more resources (Bakker and Demerouti, 2007). This conjecture also provides an important contribution for the cyberbullying literature and the role of teachers as bystanders of their students’ cyberbullying behavior, because it may lead to new clues as to why these professionals may morally disengage from these incidents, as opposed to intervene pro-socially.

### Practical Implications

The present study demonstrated the positive association of a psychosocial risk, and consequent job demand for teachers, which is observing harmful events among students (as is cyberbullying), with their productivity loss due to presenteeism and burnout levels. Accordingly, presenteeism seems to constitute an increasing health and productivity risk (Demerouti et al., 2009). Thus, it is important to manage the possible impact of observing cyberbullying among students and presenteeism with both prevention and mitigation strategies within a systemic approach. Educational systems could invest in identifying the key risk factors for teachers as bystanders of violence among their students, and as agents performing work tasks under potential psychological and physical health strain, which could potentially lead them to burnout. In turn, policy makers could emphasize laws which could reduce these risk factors, whereas parents’ associations could be sensitive to the issues surrounding teachers’ role in managing cyberbullying situations and how these could impact their wellbeing and, consequently, their performance in class. Lower quality in teaching could inevitably impact learning.

It would also be important to develop strategic training programs backed by governmental institutions and parents’ associations based on social and emotional learning strategies (Oliveira et al., 2021a) to minimize the potential impact of observing cyberbullying on teachers’ productivity loss due to presenteeism and burnout. These programs could take on a whole-institution systemic approach and could include specific and tailored strategies involving social and emotional learning core areas, such as self-awareness, self-management, social awareness, relationship skills, and responsible decision making (Durlak et al., 2015; Oliveira et al., 2021b).

Initial and in-service teacher training could consider new job demands, such as knowing how to deal with phenomena, such as cyberbullying, which have an impact on the regular functioning of students (Ferreira et al., 2020), and as this study revealed, are also positively associated with teacher productivity loss due to presenteeism and burnout. It is a risk in itself for institutions to consider that teachers have all the necessary resources to deal with such phenomena, because these events may have high health and productivity costs. It would be important to provide training opportunities for teachers to become more aware of themselves as professionals and the new possible job demands that may constitute a psychosocial risk for them and their students. Another important aspect could be the shared regulation of new job demands through collaboration with other professionals, such as the schools’ counselors/psychologists, other teachers, school assistants, and the board of directors. It is crucial to build a positive institutional climate with a support network to collaborate with ill teachers so that they may manage work issues (Dudenhöffer et al., 2017).

Furthermore, developing a culture of awareness within institutions, where professionals who may be struggling with such job demands, are identified, supported, and encouraged to take some time to recover physically and psychologically (Fritz and Sonnentag, 2005). During this time, a systemic support system could be implemented where colleagues could be compensated to temporarily cover for the teacher during his/her time of recovery. By doing so, long-term negative effects of productivity loss due to presenteeism could be avoided (Demerouti et al., 2009). Accordingly, if this is implemented on a systemic level, then, school principals could lead by example and other colleagues could be role models and develop a belief system where taking time to recover is not seen as a taboo. By implementing such practices, educational systems could avoid scenarios with more burned out teachers and even contribute to the wellbeing of school communities by providing an adaptive resolution for violent phenomena among students, such as cyberbullying. Lastly, students could also benefit from understanding how observing incidents of cyberbullying could impact their own presenteeism when in class and their levels of burnout as well. Therefore, measures to assess these variables would also be a step forward to implement wellbeing among school communities.

### Limitations and Future Directions

This study is not without limitations. It is cross-sectional in nature and therefore, it was not used to assess behavior over time and determine cause and effect among variables. Therefore, future studies could investigate the examined variables with tools that would enable them to capture objective data concerning teachers’ reactions to cyberbullying events and later performance indicators, such as serious games (Ferreira et al., 2021). Accordingly, it would also be interesting to invest in a longitudinal analysis of the interaction between the examined variables (Ruhle et al., 2020), similarly to what previous research has done with presenteeism, burnout, and other variables (Demerouti et al., 2009). This would enable future research to investigate specifically how observing cyberbullying among students could lead to productivity loss due to presenteeism but mediated by burnout – a relationship which seems to be reciprocal in specific contexts (Demerouti et al., 2009). Although the response rate in our study was 100%, because all of the teachers answered the entire protocol due to forcing response options in the online format, no researcher was present while participants were answering. Future research could provide an online survey, but within a school context with a researcher present to monitor participants and help with any technical issues. Moreover, despite our large sample, since we worked with data pertaining to cyberbullying, which may include data that deviates from normality, as it is criminal behavior, we used bootstrapping, a nonparametric resampling procedure, to account for any violations of normality assumptions. In fact, bootstrapping tests mediation without imposing the assumption of normally distributed data (Preacher and Hayes, 2008) and shows greater power while controlling the Type I error rate, which is an advantage (Preacher and Hayes, 2004). Also, although we considered productivity loss due to presenteeism, future research could also consider examining a process approach of presenteeism (Ruhle et al., 2020), focusing on individuals’ experience during the pandemic with qualitative measures. Furthermore, it would also be interesting for future research to consider other variables that teachers could potentially use and self-protection strategies to deal with observing cyberbullying and not be affected by it, such as moral disengagement mechanisms (Bandura, 2002). This study only considered a perspective on burnout as majorly emotional exhaustion (Kristensen et al., 2005); therefore, future research could consider other perspectives of the construct. Even though we provided information regarding occupational/sectoral area and broader context of a confined working environment due to the COVID19 pandemic (Ruhle et al., 2020), future research could also focus on investigating the relationship between the examined variables would also be interesting in a post-pandemic context to understand whether the relationships would still hold or be different. Other variables could be considered as important to the relationships we proposed in this study, such as teachers’ self-efficacy at work regarding task management and using problem-solving strategies in cyberbullying situations. Lastly, the fact that teachers had to adapt to a new model of teaching in a short period of time, and during a pandemic, may have also contributed to presenteeism and burnout among these professionals.

### Conclusion

During the pandemic caused by SARS-CoV2, teachers performed their tasks, despite the difficulties they faced. Addressing teachers’ wellbeing and the disturbances which may have affected their work and which emerged during the pandemic is of vital importance for a universal understanding of how educational systems dealt with the adversities (Dorcet et al., 2020). This study responded to this call and found that observing cyberbullying incidents among students can be considered a psychosocial risk and consequent job demand which was positively associated to productivity loss due to presenteeism and burnout. As cyberbullying proliferated during the COVID-19 pandemic with devastating consequences for those involved (Barlett et al., 2021), we suggest that future avenues of research and opportunities for professional training may consider the results presented here to meet further challenges that may arise at a global scale and impact worldwide educational institutions and its collaborators.

## Data Availability Statement

The raw data supporting the conclusions of this article will be made available by the authors, without undue reservation.

## Ethics Statement

The studies involving human participants were reviewed and approved by Comissão de Ética de Deontologia da Faculdade de Psicologia da Universidade de Lisboa. The patients/participants provided their written informed consent to participate in this study.

## Author Contributions

PF has first authorship. AB, NP, AP, and AS have contributed equally to this work and share second authorship. PF designed and executed the study, analyzed the data, and wrote, edited, and revised the manuscript. AB assisted with the design, data gathering, and the editing of the final manuscript. NP collaborated with data gathering, the editing, and formatting of the manuscript. AP assisted with data gathering, theoretical section, and the editing of the manuscript. AS assisted with the design of the study, data gathering, and editing of the final manuscript. All authors approved the final version of the manuscript for submission.

## Funding

This study was funded by the Portuguese Foundation for Science and Technology (PTDC/PSI-GER/1918/2020) and the Research Center for Psychological Science (UIDB/04527/2020 & UIDP/04527/2020).

## Conflict of Interest

The authors declare that the research was conducted in the absence of any commercial or financial relationships that could be construed as a potential conflict of interest.

## Publisher’s Note

All claims expressed in this article are solely those of the authors and do not necessarily represent those of their affiliated organizations, or those of the publisher, the editors and the reviewers. Any product that may be evaluated in this article, or claim that may be made by its manufacturer, is not guaranteed or endorsed by the publisher.
